# d,l-Sulforaphane Induces ROS-Dependent Apoptosis in Human Gliomablastoma Cells by Inactivating STAT3 Signaling Pathway

**DOI:** 10.3390/ijms18010072

**Published:** 2017-01-04

**Authors:** Ziwei Miao, Fei Yu, Yahao Ren, Jun Yang

**Affiliations:** 1 Department of Developmental Cell Biology, Key Lab of Cell Biology, Ministry of Public Health, Key Laboratory of Medical Cell Biology, Ministry of Education, College of Basic Science, China Medical University, No. 77 Puhe Road, Shenyang North New Area, Shenyang 110122, China; zwmiao@cmu.edu.cn; 2 Department of Nutrition and Food Hygiene, School of Public Health, China Medical University, No. 77 Puhe Road, Shenyang North New Area, Shenyang 110122, China; fyu08@cmu.edu.cn (F.Y.); yhren@cmu.edu.cn (Y.R.)

**Keywords:** glioblastoma multiforme, sulforaphane, apoptosis, reactive oxygen species, STAT3

## Abstract

d,l-Sulforaphane (SFN), a synthetic analogue of broccoli-derived isomer l-SFN, exerts cytotoxic effects on multiple tumor cell types through different mechanisms and is more potent than the l-isomer at inhibiting cancer growth. However, the means by which SFN impairs glioblastoma (GBM) cells remains poorly understood. In this study, we investigated the anti-cancer effect of SFN in GBM cells and determined the underlying molecular mechanisms. Cell viability assays, flow cytometry, immunofluorescence, and Western blot results revealed that SFN could induced apoptosis of GBM cells in a dose- and time-dependent manner, via up-regulation of caspase-3 and Bax, and down-regulation of Bcl-2. Mechanistically, SFN treatment led to increase the intracellular reactive oxygen species (ROS) level in GBM cells. Meanwhile, SFN also suppressed both constitutive and IL-6-induced phosphorylation of STAT3, and the activation of upstream JAK2 and Src tyrosine kinases, dose- and time-dependently. Moreover, blockage of ROS production by using the ROS inhibitor *N*-acetyl-l-cysteine totally reversed SFN-mediated down-regulation of JAK2/Src-STAT3 signaling activation and the subsequent effects on apoptosis by blocking the induction of apoptosis-related genes in GBM cells. Taken together, our data suggests that SFN induces apoptosis in GBM cells via ROS-dependent inactivation of STAT3 phosphorylation. These findings motivate further evaluation of SFN as a cancer chemopreventive agent in GBM treatment.

## 1. Introduction

Glioblastoma multiforme (GBM), also known as glioblastoma and grade IV astrocytoma, is the most frequent and lethal form of primary malignant central nervous system and brain tumor in adults [1]. Comprehensive treatment of GBM involves surgery followed by radiotherapy and/or chemotherapy. Despite recent advances in multimodel treatment strategies, prognosis for GBM remains disappointing with a median overall survival of approximately 15 months [2]. Current chemotherapeutic strategies for GBM are hampered by several obstacles, including drug resistance, myriad side effects, and difficulties in drug crossing the blood-brain barrier (BBB) [3,4]. Therefore, the discovery of effective therapeutic agents and strategies for targeting GBM are necessary.

Epidemiological and preclinical studies show that dietary intake of natural compounds can decrease the risk of several types of cancers [5]. Sulforaphane (1-isothiocyanato-4-(methylsulfinyl)-butane) is a natural plant compound that is widely in the cruciferous vegetables and occurs naturally as an l-isomer [6]. It is a remarkable bio-active molecule that regulates cancer initiation and progression [7]. It can affect several critical genes expression, which are involved in key processes, such as apoptotic, cell cycle, and cell invasion pathways in various types of cancer [8]. Currently its synthetic analogue d,l-sulforaphane (SFN) has been mainly studied as a promising chemopreventive agent for cancer therapy. Previous studies have revealed that SFN exhibits potent anti-carcinogenic properties in multiple in vivo and in vitro tumor models by influencing multiple intracellular signaling pathways [9,10,11,12]. The potency of SFN on suppressing cell growth is relatively more effective against prostate cells compared with the natural l-form [13]. In addition, following intraperitoneal administration SFN can rapidly pass through the BBB and accumulate in the striatum and cortex [14]. However, the effects of SFN on GBM have not been adequately investigated, and the precise molecular mechanisms by which SFN exerts anti-cancer effects on GBM remain to be thoroughly explored.

Reactive oxygen species (ROS), including superoxide anion radical, hydrogen peroxide, singlet oxygen, and hydroxyl radicals, are generally derived from cellular metabolism and serve as a mediator of cellular injury through regulating cell signaling pathway and homeostasis [15]. Excessive production of ROS especially targets various signaling pathways triggering the death of cancer cells, such as signal transducer and activator of transcription 3 (STAT3) signaling cascade [16,17,18]. STAT3 is frequently abnormally activated in various tumors and its constitutive activation directly contributes to tumor development and progression [19]. Activation of STAT3 facilitates its dimerization, which leads to nuclear translocation and binding to the promoter regions of downstream target genes. STAT3-regulated genes, include controllers of tumor cell proliferation, apoptosis, and pro-angiogenic vascular endothelial growth factor [20]. The important role of STAT3 signaling in carcinogenesis and cancer progression has led to its consideration as a potential molecular target for anti-cancer agents. Several studies have found that some anti-cancer agents could inhibit STAT3 activation by generating reactive oxygen species (ROS) [21,22,23]. In this regard, we investigated whether possible crosstalk between ROS and the STAT3 signaling pathway participated in the anti-cancer effect of SFN on GBM cells.

In this study, two classic GBM cell lines U251 and U87 were used to evaluate the anti-cancer effects of SFN and made further inroads towards comprehending the underlying molecular mechanisms. We determined that SFN exhibits a strong anti-tumor effect against GBM cells via ROS-mediated inactivation of STAT3 signaling. Our present study provides the foundation for further investigation of SFN in the clinical treatment and nutritional prevention of GBM.

## 2. Results

### 2.1. SFN Effectively Suppresses the Cell Viability of GBM Cells

**Figure 1:**
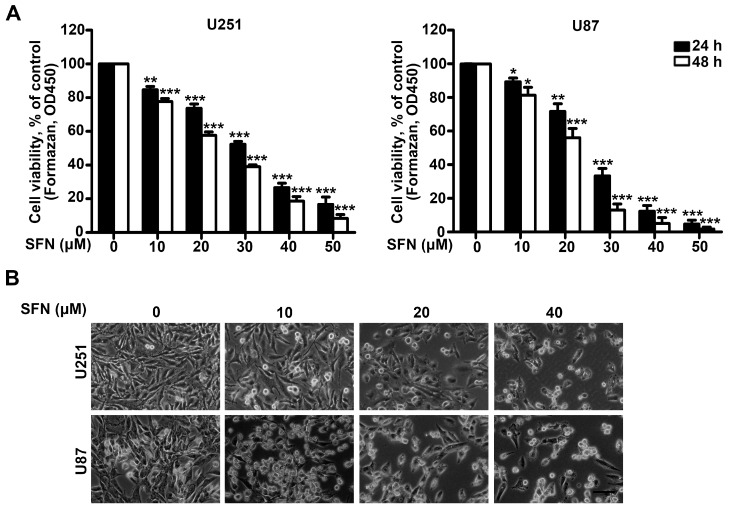
Reduced viability and morphological changes in GBM cells after SFN treatment. (**A**) Cell viability was analyzed by the CCK-8 assay when U251 and U87 cells were cultured and treated with SFN (0, 10, 20, 30, 40, 50 µM) for 24 and 48 h; and (**B**) cell morphological changes following the indicated treatment for 24 h were examined and photographed. Bar: 100 µm. Data are shown as mean ± SD. * *p* < 0.05, ** *p* < 0.01, and *** *p* < 0.001 versus control group.

To investigate the inhibitory effect of SFN on U251 and U87 GBM cells, we first analyzed the crucial role of SFN in GBM cell viability by using Cell Counting Kit-8 (CCK-8) assay. The cells were exposed to increasing concentrations of SFN for 24 and 48 h. The cell viability of the above GBM cells was significantly decreased after exposure to SFN in dose- and time-dependent manners (Figure 1A). IC_50_ values were 28.91 ± 2.54 µM for U251 cells and 26.60 ± 2.60 µM for U87 cells 24 h following treatment with SFN. In addition, we characterized the morphology of U251 and U87 cells after exposure to SFN. The results showed that SFN induced numerous dose-dependent morphological changes in both U251 and U87 cells, with loss of cell membrane asymmetry and attachment, cytoplasmic shrinkage, nuclear condensation, and filament formation (Figure 1B). These data suggest that SFN can negatively influence GBM cell viability.

### 2.2. SFN Induces Apoptotic Cell Death in GBM Cells

**Figure 2:**
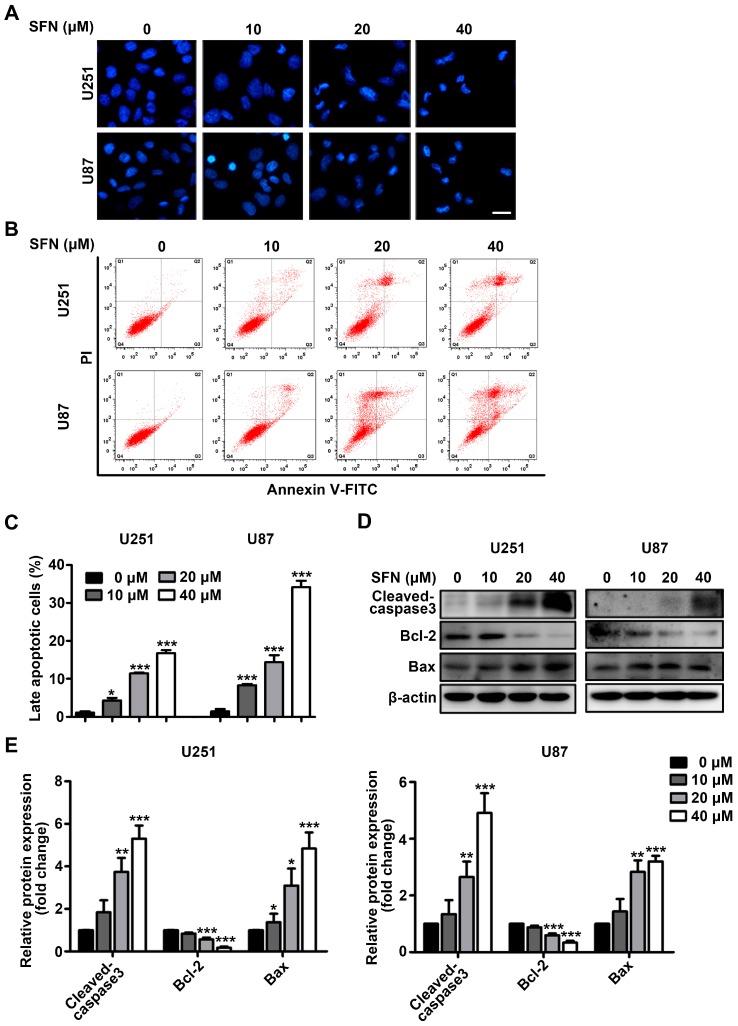
SFN induces apoptosis in GBM cells. The cells were treated with indicated concentrations of SFN for 24 h. (**A**) Nuclear morphology was observed using an inverted microscope after DAPI staining. Bar: 20 µm; (**B**) the induction of apoptosis was analyzed by double staining with Annexin V-FITC/PI, and the apoptosis rate was analyzed by flow cytometry. Cells in Q2 quarters (Annexin V positive/PI negtive) represented early apoptotic cells. The double-positive cells in Q3 quarters represented late apoptotic cells; (**C**) the percentages of late apoptotic cells relative to the total population were quantified; (**D**) Western blot analysis of cleaved caspase-3, Bcl-2, and Bax after SFN treatment; (**E**) the bands corresponding to each protein were quantified and normalized relative to control group. Data are represented as means ± SD. * *p* < 0.05, ** *p* < 0.01, and *** *p* < 0.001 versus control group.

We then determined whether the inhibition of cell viability observed following SFN treatment resulted from apoptosis. Firstly, DAPI (4′,6-diamidino-2-phenylindole) staining was employed to view the nuclear morphological characteristics after exposed U251 and U87 cells to the indicated concentrations of SFN for 24 h. Dose-dependent-chromatin condensation and nuclear fragmentation were clearly apparent in SFN-treated cells (Figure 2A). Secondly, Annexin V/PI-based flow cytometry was performed to further evaluate the pro-apoptotic effects of SFN on U251 and U87 cells by determining the numbers of the early (Annexin V positive/PI negative) and late apoptotic (Annexin V positive/PI positive) cells. As shown in Figure 2B,C, after treatment with SFN, the number of late apoptotic cells was significantly increased compared to the control cells in a dose-dependent manner. Thirdly, we detected expression changes of apoptosis-associated proteins after treatment with SFN. The Western blot results indicated that SFN could increase cleaved caspase-3 and Bax levels, and induce a concomitant decrease in the levels of Bcl-2 (Figure 2D,E). Taken together, these results indicated that SFN could induce apoptotic cell death in GBM cells.

### 2.3. ROS Generation Contributes to SFN-Induced Apoptosis of GBM Cells

**Figure 3:**
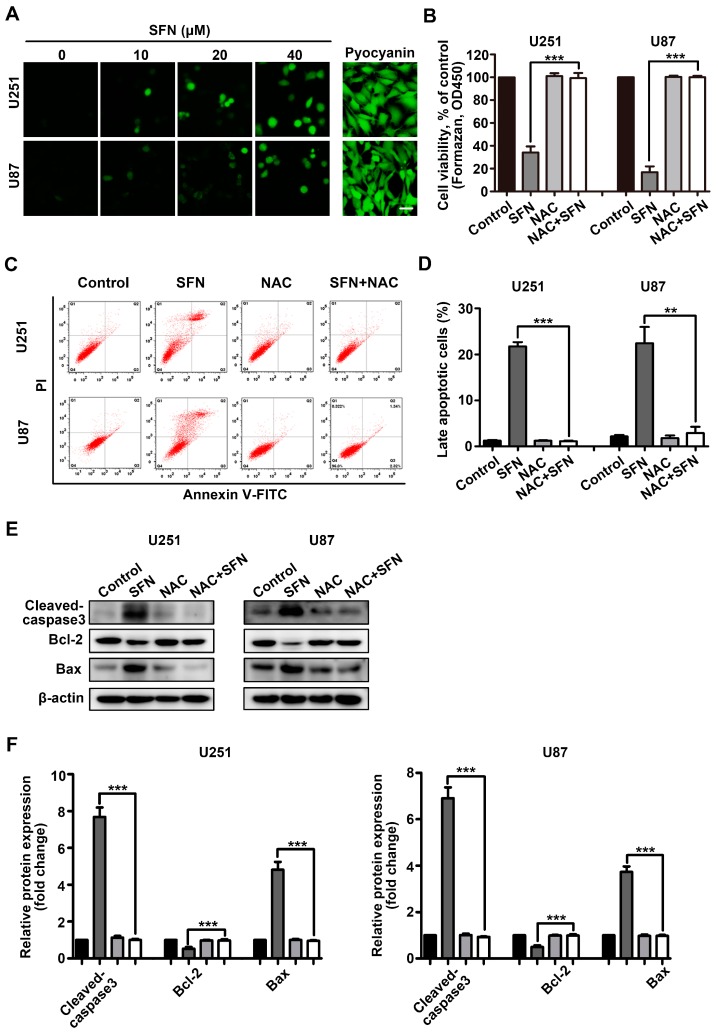
Intracellular ROS is involved in cellular apoptosis induced by SFN. (**A**) Cells were exposed to the indicated concentrations of SFN for 24 h. The level of intracellular ROS was assessed using DCFH-DA staining and observed by fluorescence microscope. As a positive control, cells were treated with pyocyanin (200 µM). Bar: 20 µm; (**B**) cells were pre-incubated with or without NAC (3 mM) for 1 h followed by the addition of SFN (40 µM) for a further 24 h. Cell viability was measured by CCK-8 assay; (**C**) cells were treated with SFN (40 µM) in the absence or presence of NAC. The apoptosis was determined by Annexin V/PI staining and analyzed by flow cytometry. Cells in Q2 quarters (Annexin V positive/PI negtive) represented early apoptotic cells. The double-positive cells in Q3 quarters represented late apoptotic cells; (**D**) the percentages of late apoptotic cells relative to the total population were quantified; (**E**) cells were treated with SFN (40 µM) for 24 h after pre-incubation with or without NAC (3 mM). The cell lysates were subjected to Western blot using anti-cleaved caspase3, anti-Bcl-2 and anti-Bax, antibodies; and (**F**) the bands corresponding to each protein were quantified and normalized relative to band intensities for control group. Data are represented as means ± SD. ** *p* < 0.01, and *** *p* < 0.001 indicates difference between SFN alone versus co-treatment with NAC.

Intracellular ROS has been generally considered to correlate with the anti-cancer activities of several nutraceuticals [24,25]. Here, we investigated whether intracellular ROS generation was involved in SFN-induced anti-cancer effects on U251 and U87 cells. Exposure to the indicated concentrations (0, 10, 20, 40 µM) of SFN dose-dependently increased the intensity of fluorescent staining signal from the specific ROS indicator DCFH-DA in U251 or U87 cells (Figure 3A). We next investigated whether ROS have a functional role in the anti-cancer effects mediated by SFN using the ROS inhibitor *N*-acetyl-l-cysteine (NAC). Pretreatment of U251 and U87 cells with NAC for 1 h significantly impaired the anti-viability effects induced by SFN (Figure 3B). Consistent results were observed when identically-treated cells were subjected to flow cytometry-based analysis of cell apoptosis (Figure 3C,D). We also used Western blot to assess the protein levels of apoptosis-related proteins following SFN treatment with NAC. NAC blocked SFN-induced changes to levels of cleaved caspase-3, Bcl-2, and Bax (Figure 3E,F). These findings suggest that SFN induced ROS accumulation and elevated levels of intracellular ROS contribute to SFN-mediated apoptosis of GBM cells.

### 2.4. SFN Attenuates the Activation of STAT3 Signaling Pathway in GBM Cells

**Figure 4:**
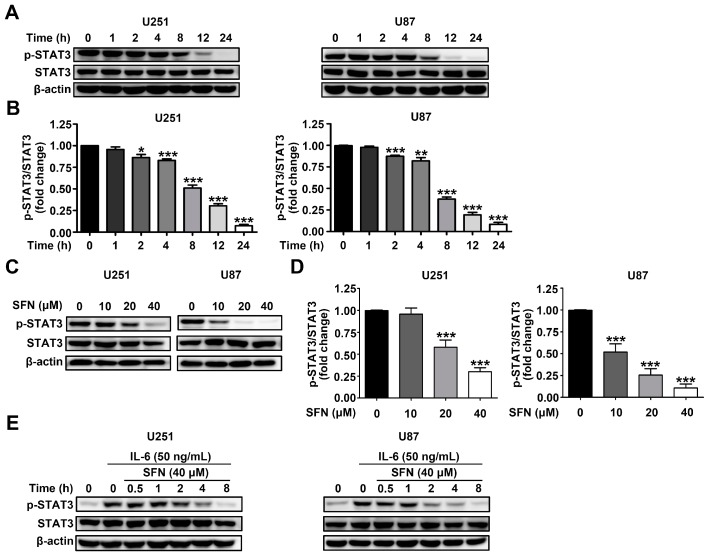

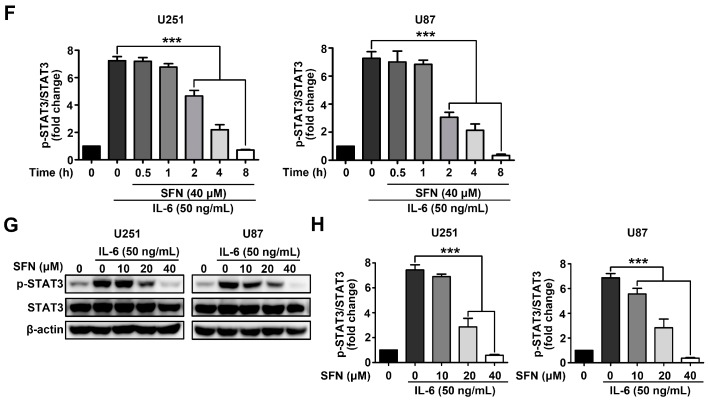
SFN effectively suppresses the activation of STAT3 in GBM cells. (**A**,**B**) Cells were treated with SFN (40 µM) and cultured for indicated time points; (**C**,**D**) cellswere treated with indicated concentrations of SFN and then harvested 24 h later; (**E**,**F**) cells were pretreated with 40 µM SFN for various time periods (0–8 h) before stimulation by IL-6 (50 ng/mL) for 15 min; and (**G**,**H**) cells were pretreated with SFN at indicated concentrations for 8 h before stimulation by IL-6 (50 ng/mL) for 15 min. Whole-cell lysates were processed for Western blot analysis using anti-phospho-STAT3 and anti-STAT3 antibodies. The bands corresponding to each protein were quantified and normalized relative to band intensities for control group. Data are represented as means ± SD. * *p* < 0.05, ** *p* < 0.01, and *** *p* < 0.001 versus the control group or the IL-6 only treatment group.

**Figure 5:**
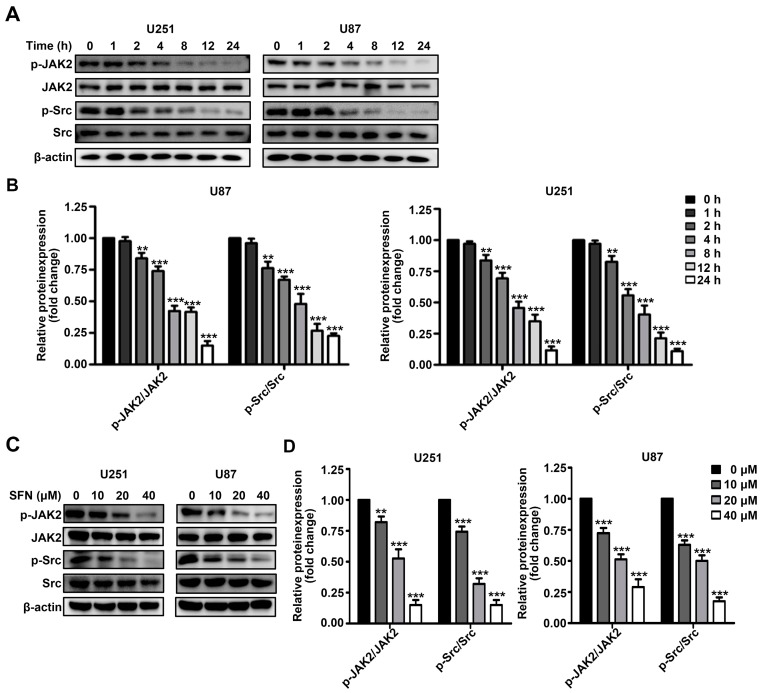
SFN inhibits phosphorylation of JAK2 and Src in GBM cells. (**A**,**B**) Cells were incubated with SFN (40 µM) for indicated time points; and (**C**,**D**) cells were treated with indicated concentrations of SFN and then harvested 24 h later. Whole-cell lysates were prepared and subjected to Western blot using anti-phospho-JAK2, anti-JAK2, anti-phospho-Src and anti-Src antibodies. The bands corresponding to each protein were quantified and normalized relative to band intensities for the control group. Data are represented as means ± SD. ** *p* < 0.01, and *** *p* < 0.001 versus control group.

The STAT3 signaling pathway plays a critical role in the modulation of various carcinogenic activities. Therefore, we examined whether SFN treatment influenced the activation of STAT3 in U251 and U87 cells. After treatment with 40 µM SFN for 0, 2, 4, 8, 12, or 24 h; or with 0, 10, 20, or 40 µM for 24 h, we monitored STAT3 activity by Western blot. The results showed a significant time-and dose-dependent reduction in phosphorylation of STAT3 after treatment with SFN in U251 and U87 cells (Figure 4A–D). Binding of the cancer-related inflammatory cytokine interleukin (IL)-6 to its receptor activates STAT3 [26]. To further characterize whether SFN could affect IL-6-induced activation of STAT3, U251 and U87 cells were treated with SFN (40 µM) for 0, 1, 2, 4, 8, 12, 24 h before exposure to IL-6 (50 ng/mL) for 15 min. Western blot analysis revealed that SFN could time-dependently inhibit STAT3 phosphorylation induced by IL-6 (Figure 4E,F). Similarly, cells pretreated with indicated concentrations (0, 10, 20, 40 µM) of SFN for 8 h followed by exposure to IL-6 (50 ng/mL) for 15 min, the data showed that SFN also exhibited dose-dependently down-regulation STAT3 activation induced by IL-6 (Figure 4G,H). As STAT3 can be activated by Janus-activated kinases (JAKs) and c-Src [27], we assessed activation of JAK2 and Src in U251 and U87 cells following SFN treatment. Our data demonstrated that exposure to SFN could time- and dose-dependently decrease the phosphorylation of JAK2 and Src in U251 and U87 cells (Figure 5A–D). These findings indicate that SFN inhibits both constitutive and IL-6-induced STAT3 phosphorylation in GBM cells.

### 2.5. ROS Generation Underlies SFN-Mediated Inactivation of STAT3 Signaling

**Figure 6:**
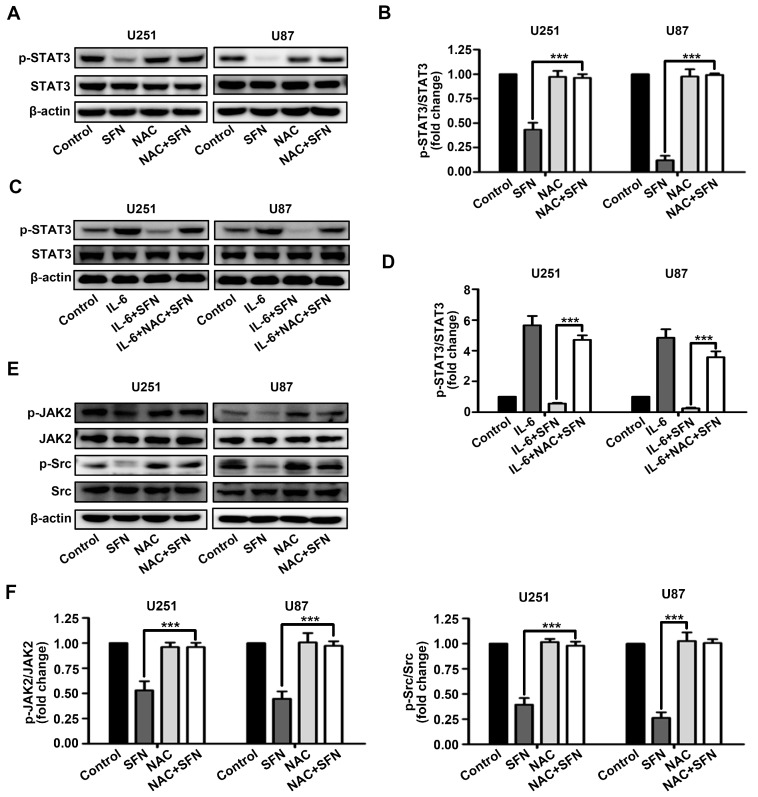
SFN-induced inactivation of STAT3 signaling in GBM cells is dependent on intracellular ROS generation. (**A**,**B**) Cells were treated with SFN (40 µM) for 24 h in absence or presence of NAC. Cell lysates were subjected to western blot to analyze the expression of phospho-STAT3 and STAT3; (**C**,**D**) cells were pre-incubated with or without NAC (3 mM), then add IL-6 (50 ng/mL) combined with SFN or DMSO. Cell lysates were subjected to Western blot to analyze the expression of phosphor-STAT3 and STAT3; and (**E**,**F**) cells were treated with SFN (40 µM) for 24 h after pre-incubation with or without NAC (3 mM). Cell lysates were subjected to Western blot to analyze the expression of phospho-JAK2, JAK2, phospho-Src, and Src. The bands corresponding to each proteins were quantified and normalized relative to band intensities for control group. Data are represented as means ± SD. *** *p* < 0.001 versus only SFN or SFN + IL-6 group.

Increased intracellular ROS levels is found to be correlated with activation of STAT3 signaling pathway. We investigated whether ROS is partly responsible for SFN-associated inhibition of STAT3 activation in U251 and U87 cells. As shown in Figure 6A,B, pretreatment with *N*-acetyl-l-cysteine (NAC), ROS scavenger, markedly blocked STAT3 dephosphorylation following SFN exposure in U251 and U87 cells. Moreover, pretreatment with NAC dramatically attenuated the activity of SFN to repress IL-6-induced STAT3 activation (Figure 6C,D). Additionally, SFN-induced dephosphorylation of JAK2 and Src was also reversed by NAC pretreatment in both U251 and U87 cells (Figure 6E,F). Collectively, the above results indicate that ROS accumulation function upstream of inactivation of the STAT3 signaling pathway induced by SFN in GBM cells.

## 3. Discussion

In the present study, we demonstrated that SFN reduced the cell viability and promoted apoptosis in GBM cells and that this effect is mediated by elevated ROS levels, which suppress activation of the STAT3 signaling cascade.

Several studies have reported that SFN has cytotoxic potential and apoptotic activity in several types of cancer. Interestingly, several normal epithelial cells are relatively resistant to apoptosis induction by SFN at concentrations to which cancer cells are death [28,29]. These make SFN well suited for cancer therapy. However, its effects on in GBM have not been explored. In this current study, we evaluated the ability of SFN to induce apoptosis and to determine the molecular mechanism underlying this process in the GBM cells. Our results show that SFN effectively induced dose- and time-dependent inhibition of the cell viability and caused morphological changes in GBM cells. Apoptosis, which regulates the homeostasis between cell proliferation and cell death, is a key mechanism by which anti-cancer agents exert their activity [30]. Several biochemical cascades are involved in the controlled execution of programmed cell death, such as pathway involving Bcl-2, Bax and caspases [31]. Therefore, we investigate the apoptosis induction in SFN-treated GBM cells. We found that SFN could induce apoptosis in GBM cells as evidenced by DAPI staining and Annexin V/PI staining. Additionally, this effect of SFN on GBM cells is further supported by the increased expression of cleaved caspase-3 and Bax, and decreased expression of Bcl-2 observed following SFN exposure. Taken together, these results suggest that SFN exerts anti-viability actions in GBM cells by promoting both cell apoptosis induction.

A better understanding of the underlying mechanisms of SFN-induced apoptosis is essential for its clinical application in GBM treatment and prevention. Studies have shown that elevated ROS production may play an important role in determining cell fate through modulation of cellular signaling pathways controlling gene expression [32]. It has been reported that ROS acts as a regulatory mediator responsible for the initiation of apoptosis. Several anti-cancer agents exhibit potent inhibitory effect on carcinogenesis via ROS-dependent activation of apoptotic cell death. Several studies show that SFN can induce cancer cell death by increasing ROS levels, however other reports reveal that SFN may protect normal cells against oxidative stress [33,34]. It has been proposed that these contradictory activities of SFN occur because cancer cells have a high inherent level of ROS, which might be activated to amplify the suicide signal induced by anti-cancer agents. In contrast, this does not happen in normal cells [35]. To verify this hypothesis, we analyzed the effect of ROS on SFN-induced apoptosis of GBM cells and showed that exposure to SFN dramatically augments the generation of intracellular ROS in a dose dependent manner. These increased levels of ROS are critical for SFN-mediated proapoptotic effects in GBM cells, as pretreatment of cells with the ROS scavenger NAC reduced the inhibitory effect on cell viability induced by SFN in GBM cells. In addition, the abrogation of ROS production by NAC totally reversed apoptosis induced by SFN in GBM cells. As expected, pretreatment of GBM cells with NAC clearly attenuated SFN-induced expression changes of apoptosis-related proteins (Bcl-2, Bax and cleaved caspase-3). These findings suggest that ROS has a significant role in SFN-induced apoptosis in GBM cells.

It is well accepted that STAT3 signaling mediates pro-proliferative signals, and modulates multiple proteins involved in the maintenance of self-renewal and tumorigenicity [36]. Several studies have shown that STAT3 signaling is constitutively activated in GBM, and chronic STAT3 activation can promote anti-apoptotic behavior, invasion, angiogenesis, and drug resistance of GBM cells and is correlated with poor clinical outcomes in GBM patients [37,38]. Moreover, blockade of activation of STAT3 signaling results in the induction of apoptosis and cell cycle arrest in GBM cells and prolonging survival in GBM xenograft tumor models [39]. It has been reported that inhibition of STAT3 partially contributes to the proapoptotic effect of SFN in prostate cancer cells [40]. Therefore, we examined whether STAT3 signaling was also involved in the anti-cancer activities upon SFN treatment in human GBM cells. The results of present study were consistent with previous findings; SFN could markedly reduce STAT3 phosphorylation in time- and dose-dependent manners. Additionally, the activation of STAT3 induced by IL-6 was also suppressed by SFN treatment. The activation of STAT3 is mediated by several upstream kinases, such as JAK2 and Src, which are activated in GBM [41,42]. Here, we show that SFN treatment could induce the time- and dose-dependent down-regulation of JAK2 and Src kinases phosphorylation in GBM cells, which is likely to inhibit STAT3. From these findings we speculated that inactivation of JAK2/STAT3 or Src/STAT3 signaling pathway may be critical for SFN-induced anti-tumor effects in GBM cells. Furthermore, we examined the crosstalk between ROS generation and STAT3 inactivation induced by SFN in GBM cells. We report the novel finding that inhibiting SFN-induced ROS generation by pre-treatment with NAC abrogated the inhibitory effects of SFN on both constitutively-active and IL-6-induced STAT3. Moreover, NAC inhibited the activation of JAK2 and Src induced by SFN. It is likely that SFN-induced ROS may inhibit JAK2 or Src phosphorylation via post-translatinal modification of cysteine residues [43]. These results suggest that SFN induces apoptosis in GBM cells by inactivating the STAT3 signaling pathway in a ROS-dependent manner.

In summary, our present study provides important information regarding the mechanisms by which SFN induces apoptosis of GBM cells. Our results demonstrate that SFN suppresses GBM cell viability by inducing apoptosis. These inhibitory effects are most likely mediated by inactivation of the STAT3 pathway via ROS. Our findings provide new insights into the function and molecular pathway to understanding the anti-cancer activity of SFN in GBM cells and support the consideration of SFN as a potent cancer chemopreventive drug candidate for the clinical treatment of GBM.

## 4. Materials and Methods

### 4.1. Reagents and Antibodies

SFN was purchased from Sigma-Aldrich (St. Louis, MO, USA) and stock solution of SFN (100 mM) was dissolved in dimethyl sulfoxide (DMSO) and diluted to the appropriate concentration in cell culture medium with a maximal DMSO concentration less than 0.5%. Pyocyanin, NAC, and DAPI were purchased from Sigma-Aldrich. Cell Counting Kit-8 (CCK-8) kit was purchased from Dojindo Laboratories (Tokyo, Japan). IL-6 was obtained from R and D Systems (Minneapolis, MN, USA). Fetal bovine serum (FBS), modified Eagle’s medium (MEM), Dulbecco′s MEM (DMEM), penicillin, and streptomycin were purchased from Invitrogen (Carlsbad, CA, USA). Antibodies against STAT3, phospho-STAT3, JAK2, phospho-JAK2, Src, phospho-Src were purchased from Cell Signaling Technology (Beverly, MA, USA). Antibodies against cleaved caspase-3, Bcl-2, Bax and β-actin were purchased from Abcam (Cambridge, UK).

### 4.2. Cell Lines and Culture

The human GBM cell lines U251 and U87 were purchased from Shanghai Institute for Biological Sciences, Chinese Academy of Sciences (Shanghai, China) and grown in DMEM or MEM, respectively. The cells were cultured with 10% FBS, 100 U/mL of penicillin, and 100 µg/mL of streptomycin in a humidified atmosphere of 95% air and 5% CO_2_ at 37 °C.

### 4.3. Cell Viability Assay

Cell viability was evaluated using the CCK-8 assay. Briefly, 1 × 10^4^ cells/per well were seeded into 96-well tissue culture plates and treated with indicated concentrations of SFN for 24 or 48 h in five parallel wells. Next, 10 µL of CCK-8 solution was diluted in the 100 µL culture medium was added to each well and cells incubated for 1 h. The concentration of the formazan generated by dehydrogenases was measured at a wavelength of 450 nm using microplate reader (Bio-Rad Laboratories, Richmond, CA, USA). The rater of cell viability is expressed as a percentage and normalized to values obtained from control cells.

### 4.4. DAPI Staining

Nuclei were stained with DAPI to evaluate chromatin condensation changes associated with apoptosis. Briefly, cells were seeded at 1 × 10^5^ cells per well in six-well tissue culture plates. GBM cells treated with the required concentrations of SFN were washed with PBS and fixed in 4% formaldehyde for 30 min. Cells were then permeabilized, washed repeatedly with PBS, and stained with DAPI solution for 5 min. After incubation, samples were mounted on a glass slide and images acquired using a BX51 fluorescence microscope (Olympus, Tokyo, Japan).

### 4.5. Apoptosis Analysis

Apoptotic cells were detected using the Annexin V-FITC Apoptosis Detection Kit (BD Biosciences, San Jose, CA, USA). Briefly, cells were seeded in six-well tissue culture plates at a density of 1 × 10^5^ cells per well. After exposure to SFN for 24 h, both attached and floating cells were collected. After washing with PBS, GBM cells were resuspended in binding buffer. Following 15 min incubation with Annexin V-FITC and propidium iodide (PI) at room temperature in the dark, stained samples were analyzed using a flow cytometer (FACScan, Becton Dickinson, Franklin Lakes, NJ, USA). Acquisition and analysis of the data were performed using FlowJo 7.6 software.

### 4.6. Measurement of Intracellular ROS Level

The levels of intracellular ROS were measured using DCFH-DA fluorescent dye (Beyotime, Nanjing, China) according to the manufacturers′ recommendations. Briefly, cells were cultured in six-well tissue culture plates and incubated with indicated concentrations of SFN for 24 h. As a positive control, the cells were exposed to pyocyanin (an ROS inducer, 200 µM) for 3 h. After treatment, cells were incubated with 10 µM DCFH-DA diluted in serum-free culture medium for 30 min at 37 °C in the dark. After incubation, the cells were washed twice with PBS and micrographs obtained using a conventional fluorescent microscope (Olympus).

### 4.7. Cell Fractionation and Western Blot Analysis

After treatment with SFN as required, cell lysates were prepared in ice-cold radio- immunoprecipitation assay (RIPA) buffer (Beyotime) containing protease inhibitors, and analyzed by Western blot as described previously [23]. Briefly, total proteins were separated by SDS-polyacrylamide gel electrophoresis. After electrophoresis, proteins were transferred to a polyvinylidene difluoride (PVDF) membrane. Then, the membrane was blocked for 1 h at room temperature, and probed with a 1:1000 dilution of indicated primary antibodies overnight at 4 °C. After exposure to HRP-conjugated secondary antibodies (1:5000; Abcam) for 1 h at room temperature, the immunostained protein bands were developed using ECL Prime Western Blotting Detection reagent (GE Healthcare, Buckinghamshire, UK), and images were obtained using an LAS3000 mini instrument (Fuji Film, Tokyo, Japan). Quantification of band density was analyzed by Image J 1.48 software. Signals were densitometrically quantified and normalized to β-actin expression.

### 4.8. Statistical Analysis

All results were obtained from at least three replicates. Statistical evaluation was performed using the Student′s *t*-test followed by one-way ANOVA to assess differences between the groups. Values are expressed as the mean ± standard error (SD). An association was considered statistically significant when *p* < 0.05.
